# Erdheim-Chester Disease: A Diagnostic Challenge

**DOI:** 10.7759/cureus.103549

**Published:** 2026-02-13

**Authors:** Teresa Soares Costa, Rita Noversa de Sousa, Margarida Oliveira, Raquel Calisto, Diana Rocha, P. Ricardo Pereira

**Affiliations:** 1 Internal Medicine, Matosinhos Local Health Unit, Matosinhos, PRT

**Keywords:** atrioventricular conduction block, erdheim-chester disease, hairy kidneys, non-langerhans cell histiocytosis, pericardial effusion, retroperitoneal fibrosis

## Abstract

Erdheim-Chester disease (ECD) is a rare, non-Langerhans cell histiocytosis characterized by excessive production and accumulation of histiocytes within various tissues. The discovery of activating mutations within the Mitogen-Activated Protein Kinase (MAPK) pathway has established its neoplastic nature and enabled the use of targeted therapeutic approaches. Despite these advances, ECD remains a diagnostic challenge, often due to its nonspecific symptoms, multisystemic involvement, and consequent delay in diagnosis. We report three cases of ECD illustrating its diverse clinical presentations and diagnostic complexity. The first case involved an elderly woman with retroperitoneal and pericardial involvement, who developed urinary sepsis and died shortly after diagnosis. The second case was a man in his seventies with cardiovascular and renal involvement who did not respond to corticosteroids or peginterferon alfa-2a and died within a year. The third case, also a man in his seventies, presented with similar multisystemic involvement but achieved clinical and radiological improvement following targeted v-Raf murine sarcoma viral oncogene homolog B1 (BRAF) inhibition. These cases highlight the heterogeneous course of ECD and the critical importance of early recognition and tailored therapy. Reporting additional cases contributes to a deeper understanding of its variable features, diagnostic pitfalls, and treatment outcomes.

## Introduction

Erdheim-Chester disease (ECD) is a rare, non-Langerhans cell histiocytosis characterized by excessive accumulation of histiocytes within tissues. ECD is in more than 80% of patients associated with mutations activating the Mitogen-Activated Protein Kinase (MAPK) pathway, mainly the v-Raf murine sarcoma viral oncogene homolog B1 (BRAF) V600E activating mutation [1,2]. While these molecular insights have improved understanding of the disease and enabled targeted therapies, the clinical diagnosis of ECD remains challenging. Its heterogeneous and often nonspecific manifestations can involve multiple organs, frequently leading to delayed diagnosis and advanced disease at presentation, which negatively impacts prognosis [3]. In this article, we describe three clinical cases of ECD with varied presentations.

## Case presentation

Case 1 

**Figure 1:**
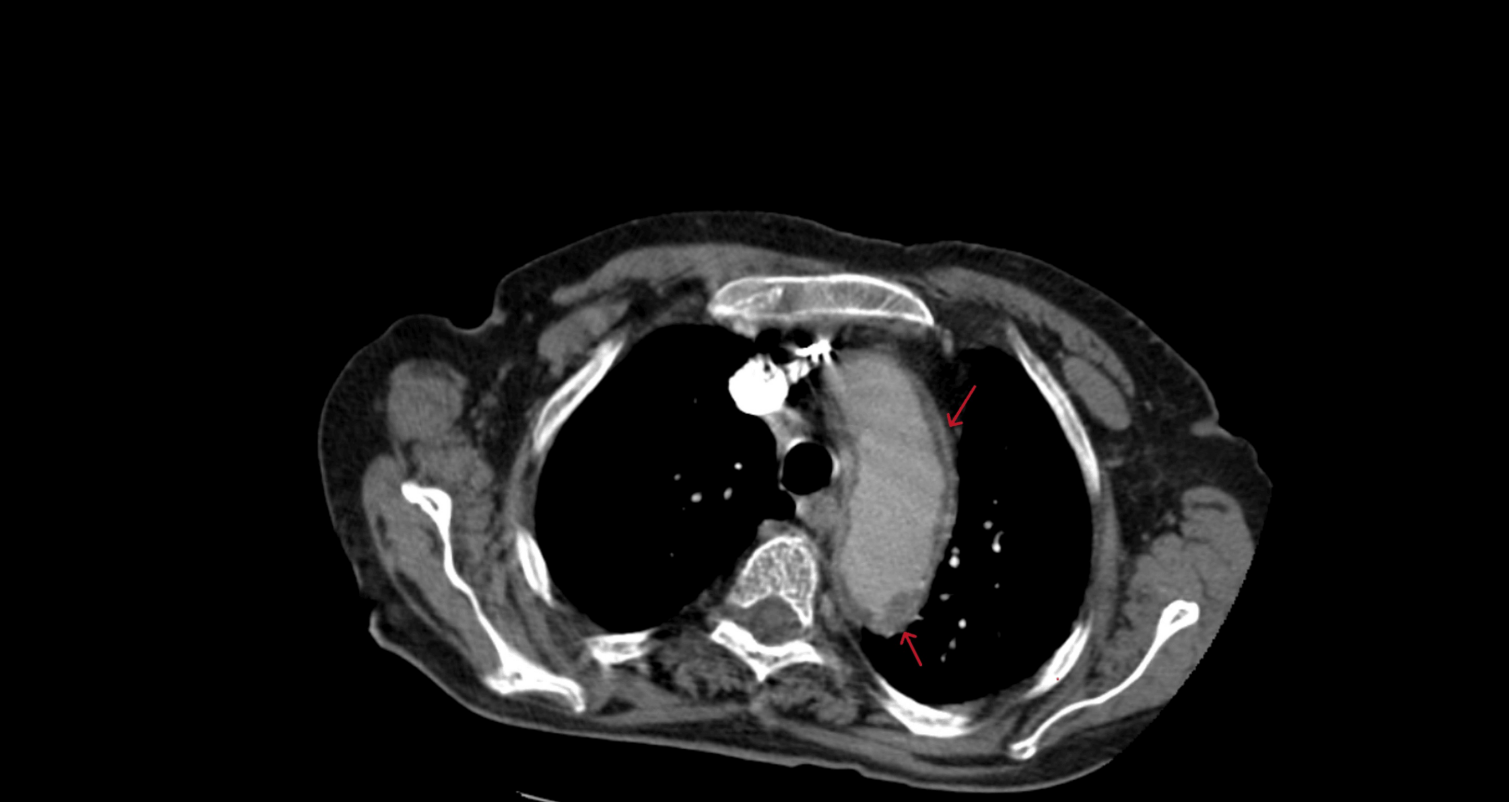
Computed tomography image of the chest (axial view) CT image showing circumferential infiltration of the aorta, known as “coated aorta” (red arrows).

**Figure 2:**
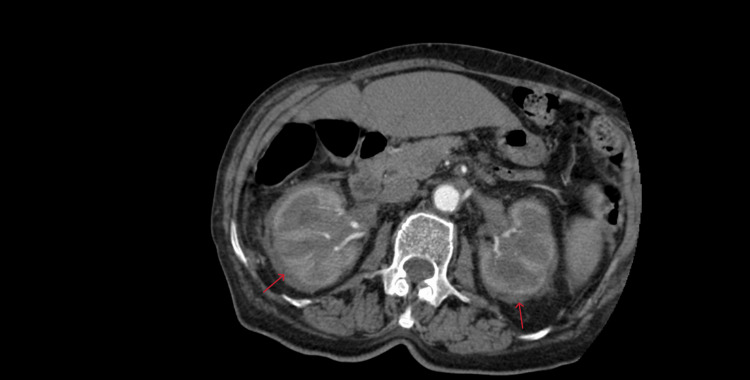
computed tomography scan of the kidneys (axial view) CT showing bilateral perirenal soft tissue halo.

**Figure 3:**
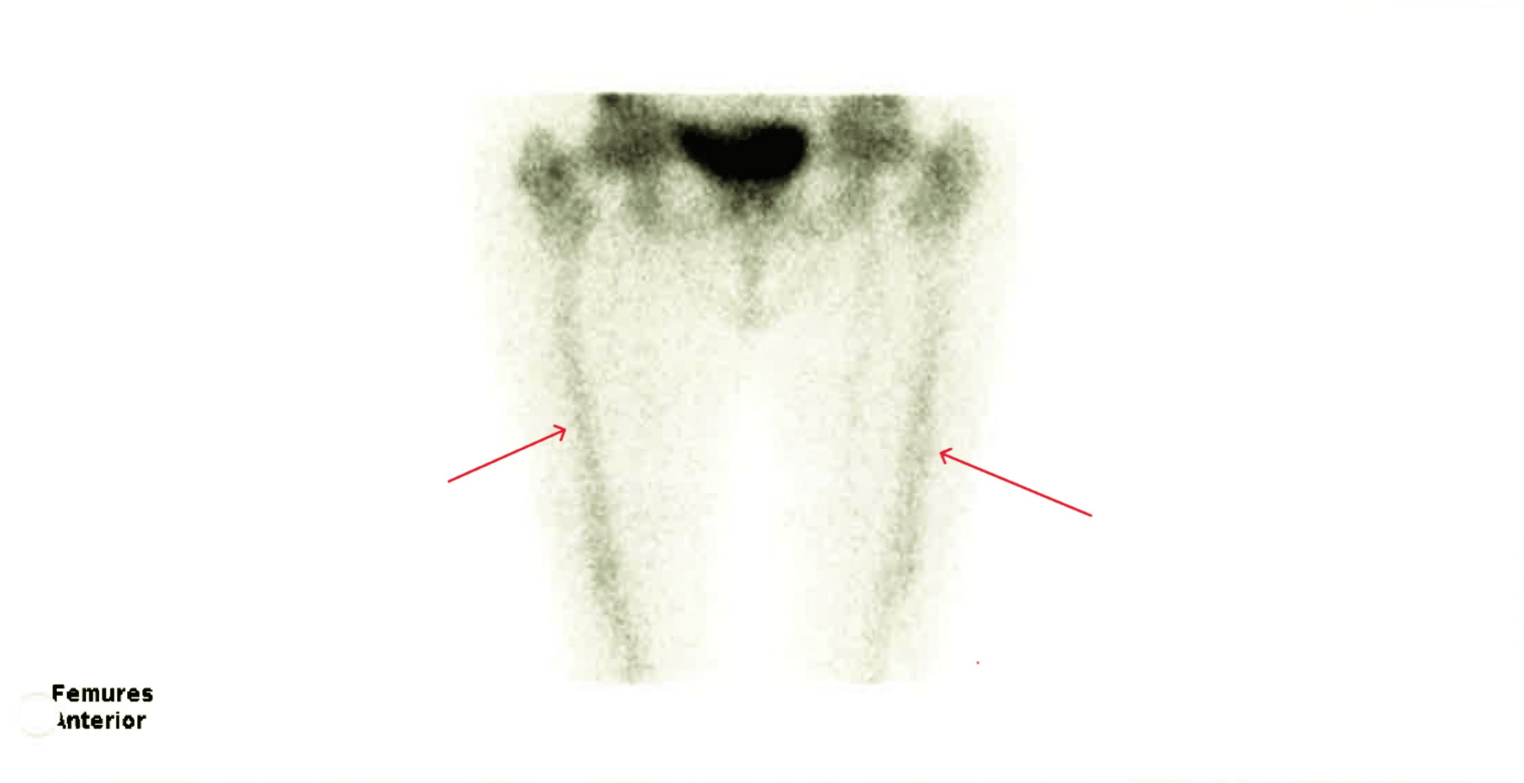
Bone scintigraphy of the femurs Bone scintigraphy showed symmetric, discrete, and diffuse increased radiopharmaceutical uptake along the diaphysis of both femurs. The red arrows show an increased uptake in femurs.

A woman in her 80s presented with self-limited retrosternal chest pain, paroxysmal nocturnal dyspnea, and weight loss (10% in two months). She had a significant medical history of essential thrombocytosis (JAK2 heterozygotic mutation). She presented with hypophonic sounds upon auscultation and an elevated jugular venous pulse to 12 cm of water above the right atrium. Laboratory tests revealed thrombocytosis, leukocytosis, and C-reactive protein levels of 65 mg/L. An echocardiogram demonstrated a moderate-volume pericardial effusion, without tamponade. Pericardiocentesis was not possible due to the posterior location of the effusion. Autoimmune and paraneoplastic causes were excluded. A computerized-tomography (CT) showed a “coated aorta” (Figure 1), pericardial effusion, and bilateral perirenal soft tissue halo (Figure 2). Bone scintigraphy showed increased radiopharmaceutical uptake along the diaphysis of the femurs (Figure 3). Perirenal mass biopsy revealed infiltration by foamy cytoplasmic macrophages (CD68 positive), compatible with ECD. Genetic study of the mass cells did not find the p.Val600Glu pathogenic variant in the BRAF gene. The patient experienced functional decline due to urinary sepsis, ultimately resulting in the patient's death [4].

Case 2

A man in his early 70s, with no relevant past medical history, presented with worsening dyspnea at rest, orthopnea, and paroxysmal nocturnal dyspnea. He was diagnosed with second-degree atrioventricular block (Mobitz II) and subsequently underwent pacemaker implantation. He also reported a 20 kg weight loss over the past one to two years. Laboratory tests indicated microcytic anemia, leukocytosis with neutrophilia, thrombocytosis, and a C-reactive protein of 54.5 mg/L. An echocardiogram showed severe left atrial dilation, moderate aortic regurgitation, and mild left ventricular dilation. CT showed mediastinal and periaortic densification from the ascending to abdominal aorta (Video 1), with bilateral perirenal involvement and symmetrical proximal humeral metaphyseal heterogeneities.

**Video 1:** A series of computed tomography scan images of thorax (axial views) Sequential axial computed tomography images of the chest demonstrating bilateral pleural and fissural loculated (encysted) effusions and extensive mediastinal soft-tissue densification. The soft-tissue involvement extends along the ascending aorta, aortic arch, descending thoracic aorta, and the origin of the iliac trunks, resulting in rightward displacement of the trachea and anterior displacement of the thoracic esophagus and carina, with bilateral extension into the paravertebral spaces.

The patient underwent biopsy of the mediastinal mass, revealing diffuse infiltration by macrophages with foamy cytoplasm, with positivity for CD68. BRAF gene mutation analysis revealed the c.1799T>A p.(Val600Glu) mutation, corresponding to the p.(V600E) variant. A positron emission tomography scan (PET) reported slight hypermetabolic involvement in the soft tissue densification in the middle and posterior mediastinum, near the left renal fascia, the celiac trunk and superior mesenteric artery, and at the medullary/bone level in the femurs and tibias.

Despite initial treatment attempts with corticosteroids and subsequently peginterferon alfa 2a, the patient did not improve. A therapeutic escalation to vemurafenib was considered; however, it could not be implemented due to progressive clinical deterioration. Overall, the unfavorable outcome was primarily driven by advanced, multisystemic disease with rapid clinical progression. Furthermore, poor adherence to treatment was observed, which may have further limited therapeutic effectiveness. Approximately one year later, the patient died.

Case 3

A man in his 70s was referred due to dyspnea over the previous year and mild precordial pain. The physical examination revealed no abnormalities. Laboratory studies revealed thrombocytosis and elevated C-reactive protein (44 mg/L), as well as sedimentation rate (43 mm/hr). The infectious and immunological study was negative. An echocardiogram showed a moderate to large volume pericardial effusion. A CT revealed a moderate-volume pericardial effusion, small pleural effusion bilaterally, and densification of the perirenal and peri-aortic tissues and dense sclerotic changes of the skeletal elements.

**Figure 4:**
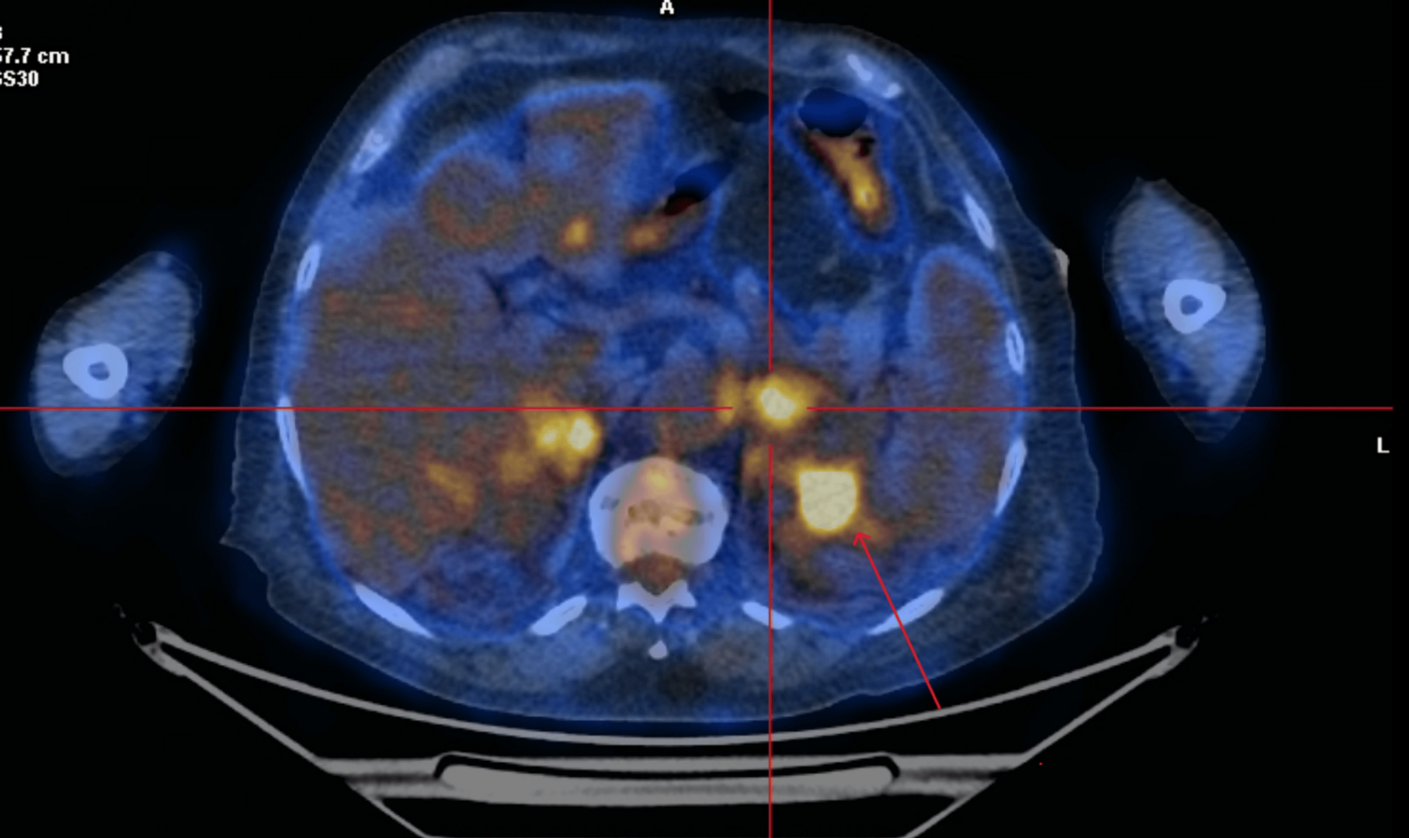
PET CT scan image of the kidney (axial view) Positron emission tomography (PET) CT scan image of the kidneys showing thickening of soft tissues in the region of the renal fascia.

**Figure 5:**
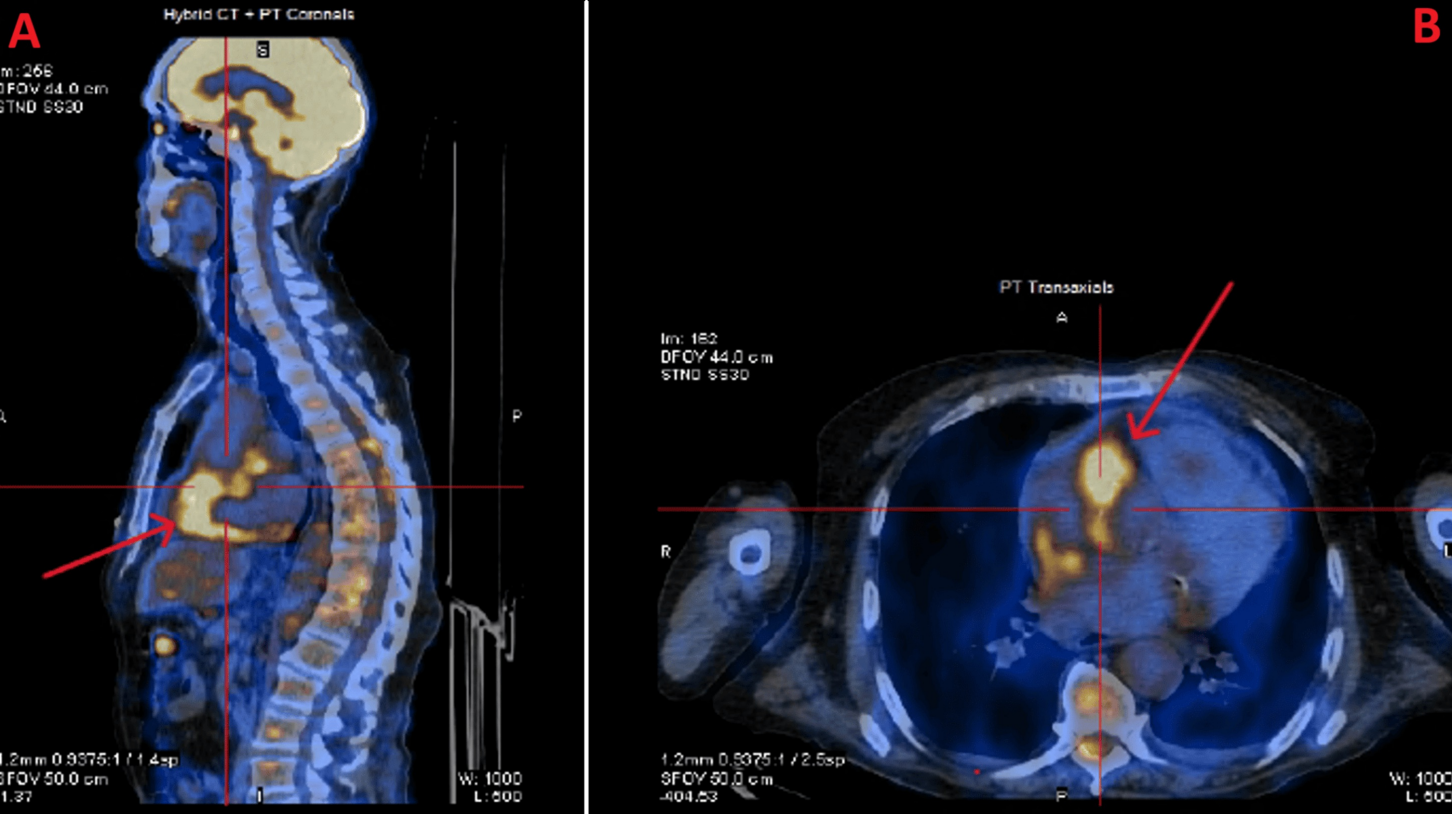
Fused PET scan images demonstrating hypermetabolic soft-tissue thickening in the pericardial region (A) Sagittal Positron emission tomography (PET) scan image of the thorax, where the red arrow shows thickening of soft tissues in the region near the heart and pericardium. (B) Axial PET scan image of the thorax, where the red arrow shows thickening of soft tissues in the region near the heart and pericardium.

A pericardiocentesis was performed, with drainage of 1 liter of pericardial fluid, which on analysis revealed a chronic inflammatory effusion without malignant cells. Perirenal biopsy analysis revealed an inflammatory infiltrate of lymphohistiocytic predominance with positivity for CD68. The genetic study detected the p.Val600Glu mutation in the BRAF gene. A PET scan showed thickening of soft tissues in the region of the renal fascia (Figure 4), abdominal aorta, and adrenal glands, and near the heart, pericardium, and thoracic aorta (Figure 5). Bone scintigraphy revealed hyperfixation in the femurs, tibias, tarsi, and humeral heads. Brain and cardiac MRI showed no features of ECD involvement. Vemurafenib was initiated, with clinical and radiological improvement being observed. An initial typical cutaneous reaction was successfully managed, allowing for gradual titration to the full dose (960 mg twice daily).

Table 1 shows a summary of all three cases reported in this article.

**Table 1:** Summary of the three reported cases BRAF: v-Raf murine sarcoma viral oncogene homolog B1.

Feature	Case 1	Case 2	Case 3
Age/Sex	88 years old/Feminine	74 years old/Masculine	76 years old/Masculine
Symptoms	Chest pain, weight loss	Dyspnea, weight loss	Dyspnea, orthopnea
BRAF Status	Negative	V600E positive	V600E positive
Treatment	Supportive care	Corticosteroids, interferon	Vemurafenib
Outcome	Death (sepsis)	Death (disease progression)	Clinically improved

## Discussion

ECD is an orphan disorder characterized by the pathological accumulation of foamy histiocytes in various organs. Studies have shown that it is often associated with a somatic mutation in the BRAF gene that causes activation of histiocytes [5]. Bone involvement is the most common feature, and the hallmark radiological feature is bilateral symmetric osteosclerosis of the long bones. Diabetes insipidus and proptosis are also common; however, none of the patients in this case series exhibited these conditions [6]. Cardiovascular involvement can include periaortic fibrosis ("coated aorta"), valvular involvement, pericardial effusion, and myocardial infiltration. Retroperitoneal manifestations, such as perirenal fat infiltration ("hairy kidneys"), are also common [7-8]. Imaging studies, including CT and PET scans, play a crucial role in identifying the extent of organ involvement [9-10].

Histological examination with immunohistochemical staining is essential, typically revealing a xanthogranulomatous infiltration of foamy CD68+, CD163+, CD1a-, S100- histiocytes. Since 2012, recurrent mutations and fusions in the MAPK (RAS-RAF-MEK-ERK) and PI3K-AKT pathways have been identified in some ECD patients. These findings confirmed that ECD is a clonal neoplastic disease driven by constant MAPK signaling and led to the development of targeted molecular therapies [3].

The coexistence of histiocytic disorders with myeloproliferative neoplasms, most notably JAK2 V617F-positive essential thrombocythemia, has been reported in several cases and raises the possibility of a shared hematopoietic origin. This hypothesis is further supported by the identification of shared mutations (JAK2, BRAF, MAP2K1) across both conditions [11]. Case 1 exemplifies this potential biological connection, describing a patient with ECD and heterozygous JAK2-mutated essential thrombocythemia. Indeed, a study with clinical and molecular analysis of 120 patients with ECD concluded that 42.5% exhibited clonal hematopoiesis, while 15.8% developed a hematologic malignancy, mostly of myeloid origin [12].

Conventional therapies have been employed over the years, and studies have shown that IFN-α or PEG-IFNα significantly improve survival. In patients with the BRAF V600E mutation, such as the patient in the third case report, treatment with BRAF inhibitors, such as vemurafenib, has demonstrated promising results [11]. In contrast, patients without access to targeted therapy or without actionable mutations often exhibit poorer clinical outcomes, as illustrated by Cases 1 and 2, which followed a rapidly progressive and ultimately fatal course despite conventional treatment. The growing body of evidence for activating mutations along the MAPK-ERK pathway in non-BRAF-V600E ECD has prompted increased interest in targeting downstream components of this pathway. Although the optimal dosing and duration of targeted therapies remain undefined, emerging data suggest that intermittent dosing strategies may be useful [3,13].

## Conclusions

This case series highlights the importance of considering ECD in patients presenting with unexplained systemic symptoms and characteristic imaging findings. Although the clinical presentation can sometimes appear acute or subacute, ECD is generally a slowly progressive disease. Its rarity and the nonspecific nature of early symptoms often delay diagnosis, with many patients only coming to clinical attention after significant organ involvement. Early recognition is important because timely interventions can improve outcomes and reduce morbidity. In our series, one patient had the BRAF V600E mutation and was treated with vemurafenib, showing the benefits of targeted therapies. These cases underscore both the heterogeneity of ECD and the importance of tailoring therapy based on molecular findings and clinical presentation.
